# Risk Factors for Hypogonadism in Male Patients with Type 2 Diabetes

**DOI:** 10.1155/2016/5162167

**Published:** 2016-02-23

**Authors:** Rendong Zheng, Lin Cao, Wen Cao, Xiaoqiu Chu, Yongxin Hu, Huifeng Zhang, Juan Xu, Hongping Sun, Weiping Bao, Kemian Liu, Chao Liu

**Affiliations:** ^1^Department of Endocrinology and Metabolism, Affiliated Hospital of Integrated Traditional Chinese and Western Medicine, Nanjing University of Chinese Medicine, 100 Shizi Street, Hongshan Road, Nanjing, Jiangsu 210028, China; ^2^Department of Endocrinology and Metabolism, Jiangsu Province Academy of Traditional Chinese Medicine, 100 Shizi Street, Hongshan Road, Nanjing, Jiangsu 210028, China

## Abstract

*Background*. Male hypogonadism is an endocrine disease characterized by low levels of serum testosterone and is closely related to the development of diabetes. The purpose of the present study was to observe the risk factors for hypogonadism in male patients with type 2 diabetes.* Methods*. A total of 213 patients with type 2 diabetes were enrolled and divided into a low total testosterone (TT) group (=75) and a normal TT group (=138). The patients' blood glucose, blood lipids, serum insulin, and sex hormones were measured. The correlations between the patients' metabolic index and sex hormone levels were analyzed.* Results*. Compared with the normal TT group, body mass index (BMI), fasting insulin (FINS), and HOMA insulin resistance index (HOMA-IR) levels were significantly higher, but the luteinizing hormone (LH) levels were significantly lower in the low TT group (*p* < 0.05). Correlation analyses found that TT was negatively correlated with BMI, waist circumference (WC), FINS, and HOMA-IR. TT was positively correlated with LH and follicle-stimulating hormone (FSH).* Conclusions*. Several risk factors of diabetes associated closely with hypogonadism. BMI, metabolic syndrome (MS), HOMA-IR, and LH are independent risk factors for hypogonadism in male patients with type 2 diabetes.

## 1. Introduction

Male hypogonadism is a common disease characterized by certain clinical features and low levels of serum testosterone [1]. Its typical clinical manifestations include physical decline, memory loss, difficulty paying attention, depression, loss of libido, and erectile dysfunction. It significantly impacts patients' quality of life [2]. Decreases in testosterone levels can lead to different degrees of pathophysiologic change in bone, muscle, fat, and the cardiovascular system [3–5]. Studies have found that male hypogonadism is caused by a variety of chronic diseases [6–8]. In recent years, studies have shown that hypogonadism is closely related to the development of diabetes [9, 10]. It has been confirmed that male patients with type 2 diabetes are significantly more likely to develop hypogonadism: the proportions of diabetes patients with low total testosterone (TT) levels are 36.5% [11].

So far, it is unclear which correlates of diabetes are associated with hypogonadism. Therefore, it is especially important to explore the risk factors for hypogonadism to facilitate prevention, diagnosis, and early treatment. The aim of this study was to observe the correlation between diabetes and risk factors for male hypogonadism and to establish whether these risks factors for hypogonadism are independent of each other in male patients with type 2 diabetes.

## 2. Patients and Methods

### 2.1. Patients

Two hundred and thirteen male patients with type 2 diabetes were recruited from January 2013 to June 2015 in the Department of Endocrinology and Metabolism. Informed consent was obtained from all patients, and the study was approved by the ethics committee of the Jiangsu Province Hospital on Integration of Chinese and Western Medicine, Nanjing University of Traditional Chinese Medicine. Among diabetic patients, 95 patients (44.6%) were with normal weight, 68 patients (31.9%) with overweight, and 50 patients (23.5%) with obesity. The International Society of Andrology recommends that hypogonadism be diagnosed if TT levels are below 12 nmol/L in males [12]. All patients were divided into either a low testosterone group (TT ≤ 12 nmol/L, 75 cases) or a normal testosterone group (TT > 12 nmol/L, 138 cases). The following were exclusion criteria: (1) acute or chronic liver or kidney failure; (2) heart failure; (3) acute complications of diabetes; (5) a history of sex gland disease; (6) infectious and autoimmune disease.

### 2.2. Methods

In the measurement of general patient characteristics, the patients' height, weight, blood pressure, and waist circumference (WC) were measured. To calculate body mass index (BMI), we used the following formula: weight (kg)/height (m)^2^.

In the measurement of biochemical indicators, the patients were tested in the morning (between 6:00 and 7:00 AM) after fasting 8–10 h. A range of biochemical indicators were measured, including fasting blood glucose (FBG), glycosylated hemoglobin (HbA1c), liver and kidney function, cholesterol (TC), triglyceride (TG), low-density lipoprotein cholesterol (LDL-c), and high-density lipoprotein cholesterol (HDL-c). Blood glucose was determined by the glucose oxidase method, and blood lipid composition was determined by enzymatic analysis (Roche Diagnostics, Mannheim, Germany).

In the measurement of insulin and sex hormones, the patients were tested in the morning (between 6:00 and 7:00 AM) after fasting 8–10 h. Fasting insulin (FINS) was measured by chemiluminescence. Sex hormones including testosterone (TT), sex hormone binding globulin (SHBG), progesterone (P), prolactin (PRL), luteinizing hormone (LH), follicle-stimulating hormone (FSH), estradiol (E2), and dehydroepiandrosterone (DHEA) were also measured by chemiluminescence (Siemens Healthcare Diagnostics, New York, USA). The HOMA insulin resistance (HOMA-IR) index was calculated according to the standard formula (fasting plasma glucose × fasting plasma insulin/22.5) [13].

### 2.3. Statistical Analysis

All values are expressed as the mean ± SD for the quantitative variables and as a percentage for the qualitative variables. The characteristics of the two groups were compared by *t*-test or nonparametric Mann-Whitney *U* test for the quantitative variables and Fisher's exact test or *χ* ^2^ test for the qualitative variables. One-way ANOVA were used to analyze the difference in multiple groups. Factors associated with hypogonadism were estimated using univariate analysis by logistic regression. Data analysis was performed using SPSS 16.0, and *p* values < 0.05 were considered statistically significant.

## 3. Results

### 3.1. Comparison of General Characteristics in the Two Groups

Compared with the normal TT group, BMI and WC were increased in the low TT group (*p* < 0.05), but the age, duration of diabetes, SBP, DBP, and rate of complications of diabetes did not differ significantly (*p* > 0.05). Comorbidities of diabetes other than nonalcoholic fatty liver disease (NAFLD) and overweight or obesity also did not differ significantly (*p* > 0.05) (Table 1).

### 3.2. Comparison of Glucose, Lipid, Insulin, and HOMA-IR in the Two Groups

Compared with the normal TT group, FINS and HOMA-IR levels were higher in the low TT group (*p* < 0.05), but FBG, HbAlc, TC, TG, and HDL-c levels did not differ significantly (*p* > 0.05) (Table 2).

### 3.3. Comparison of Sex Hormones in the Two Groups

As shown in Table 3, compared with the normal TT group, TT, LH, FSH, and SHBG levels were significantly lower in the low TT group (*p* < 0.05); P, PRL, DHEA, and E2 levels were not significantly different (*p* > 0.05).

### 3.4. Comparison of TT Level and Incidence of Hypogonadism across Levels of BMI, HOMA-IR, and LH

**Figure 1:**
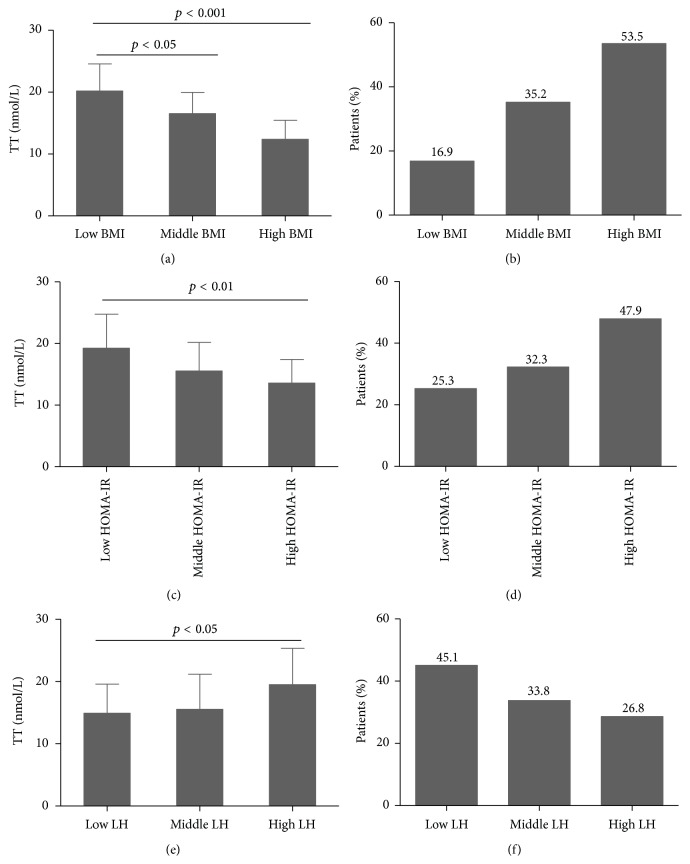
Comparison of TT concentration and percentage of patients among various groups. (a) and (b) Comparison of TT concentration and percentage of patients with hypogonadism among various BMI groups; (c) and (d) comparison of TT concentration and percentage of patients with hypogonadism among various HOMA-IR groups; (e) and (f) comparison of TT concentration and percentage of patients with hypogonadism among various LH groups.

All cases were divided into three groups according to BMI percentile (low BMI group = 0–33.3 percentile, middle BMI group = 33.4–66.7 percentile, and high BMI group = 66.8–100 percentile). Compared with the low BMI group, TT concentration was lower and the incidence of hypogonadism was higher in the high BMI group (*p* < 0.05) (Figures 1(a) and 1(b)).

In the same way, all cases were divided into three groups according to HOMA-IR percentile. Compared with the low HOMA-IR group, TT concentration was lower and the incidence of hypogonadism was higher in the high HOMA-IR group (*p* < 0.05) (Figures 1(c) and 1(d)).

In the same way, all cases were divided into three groups according to LH percentile. Compared with the high LH group, TT concentration was lower and the incidence of hypogonadism was higher in the low LH group (*p* < 0.05) (Figures 1(e) and 1(f)).

### 3.5. The Relationship between Metabolic Syndrome and TT Levels

**Figure 2:**
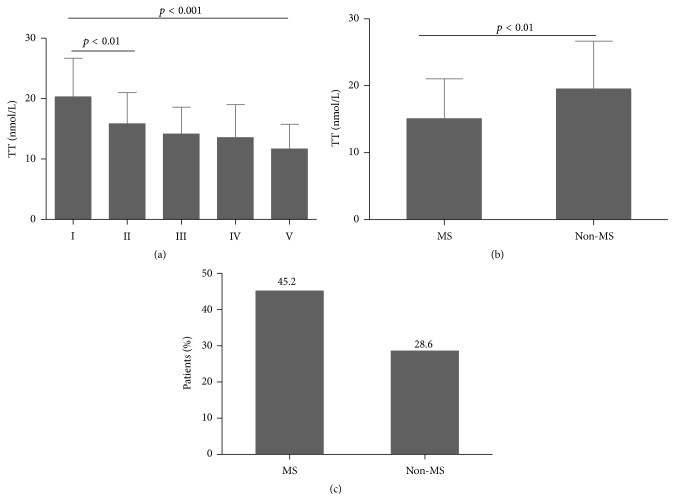
The relationship between TT concentration and MS. (a) Comparison of TT concentration among various groups. Group I, diabetes; group II, diabetes + overweight or obesity; group III, diabetes + overweight or obesity + hypertension; group IV, diabetes + overweight or obesity + hypertension + hypertriglyceridemia; group V, diabetes + overweight or obesity + hypertension + hypertriglyceridemia + low HDL-c; (b) comparison of TT concentration between MS groups and non-MS group; (c) comparison of percentage of patients with hypogonadism between MS groups and non-MS group.

Patients with 3 of the 5 characteristics identified in the ATPIII standards may be diagnosed with MS (hyperglycemia or diabetes, overweight or obesity, hypertension, hypertriglyceridemia, and low HDL-c) [14]. Patients with more characteristics of MS had lower TT concentration (*p* < 0.01) (Figure 2(a)). Compared with patients without MS, TT concentration was significantly lower and the incidence of hypogonadism was higher in patients with MS (*p* < 0.05) (Figures 2(b) and 2(c)).

### 3.6. Correlation and Regression Analysis

**Figure 3:**
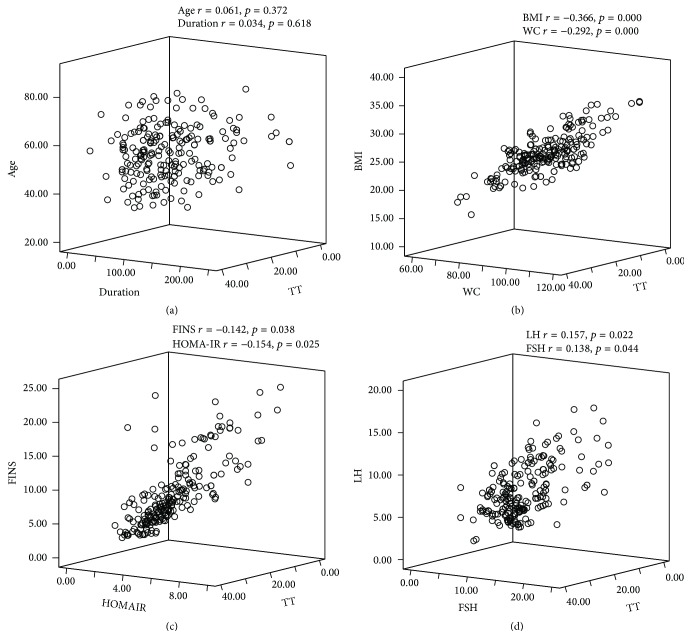
The correlation of serum TT with age and duration (a), BMI and WC (b), FINS and HOMA-IR (c), and LH and FSH (d).

Pearson correlations showed that TT was negatively correlated with BMI, WC, FINS, and HOMA-IR (*r* = −0.366, *p* = 0.00; *r* = −0.292, *p* = 0.00; *r* = −0.142, *p* = 0.038; *r* = −0.154, *p* = 0.025, resp.), while TT was positively correlated with LH and FSH (*r* = 0.157, *p* = 0.022; *r* = 0.138, *p* = 0.044, resp.). However, TT was not correlated with age or duration of diabetes (*r* = 0.061, *p* = 0.372; *r* = 0.034, *p* = 0.618, resp.) (Figures 3(a)–3(d)).

A multiple logistic regression was then run treating hypogonadism as the dependent variable and age, duration of diabetes, BMI, WC, FBG, HbA1c, FINS, HOMA-IR, TG, HDL-c, LH, FSH, and MS as independent variables. In this analysis, BMI, HOMA-IR, LH, and MS were significant independent risk factors for hypogonadism; MS was associated with hypogonadism after controlling for BMI, and HOMA-IR and LH were associated with hypogonadism after controlling for BMI and MS (Table 4).

## 4. Discussion

Type 2 diabetes can lead to organ damage throughout the body. In addition to chronic complications, diabetes is related to hypogonadism, nonalcoholic fatty liver disease, osteoporosis, cancer, and so forth. Male hypogonadism seriously affects the quality of life in patients with diabetes [15, 16].

At present, the serum total testosterone level is regarded as the most reliable indicator of hypogonadism. The International Society of Andrology recommends that hypogonadism be diagnosed if TT levels are below 12 nmol/L in males [12]. However, so far, the relationship between low testosterone levels and type 2 diabetes is still not very clear [17]. One clinical study found that patients with type 2 diabetes are prone to hypogonadism [18]. A cross-sectional study of 1089 patients with type 2 diabetes found that 36.5% of diabetes patients exhibit hypogonadism [11]. We investigated 213 patients in the present study, of which 75 cases (35.2%) had TT < 12 nmol/L, and also found that 22 cases (44.0%) had TT < 12 nmol/L in patients with obesity (50 cases). These numbers indicate that obesity patients with type 2 diabetes frequently meet the criteria for hypogonadism.

It is especially important to establish the correlation between hypogonadism and the characteristics of diabetes. It is well established that obesity is a major risk factor for type 2 diabetes and cardiovascular disease. Studies have found a positive relationship between hypogonadism and overweight or obesity [19, 20]. Our study demonstrated that serum TT showed a significant linear decrease as BMI increased, indicating that patients with type 2 diabetes and overweight or obesity often exhibit hypogonadism. A possible explanation for the significant effect of BMI on hypogonadism in men with type 2 diabetes might be that the transformation of testosterone into estrogen by aromatase results in decreases in the testosterone levels [21].

In addition, we did not find a relationship between testosterone and TG or HDL-c in our study. However, the effect of dyslipidemia on testosterone remains unexplored. One study reported that testosterone also increases the expression of hepatic lipase in HepG2 cells. Those data suggest that testosterone regulates HDL cholesterol level, promotes reverse cholesterol transport, and exerts an antiatherogenic effect [22].

Additionally, no correlation between TT and blood glucose (HbA1C) was found. FINS and HOMA-IR levels increased in the low testosterone group, and TT is negatively related to FINS and HOMA-IR. In addition, TT decreased significantly in the high HOMA-IR group. Our results show that increased insulin resistance is associated with an increased severity of hypogonadism. This indicates that insulin resistance is involved in the pathogenesis of hypogonadism in patients with type 2 diabetes. As for possible mechanisms, long-term hyperglycemia and insulin resistance affect the synthesis and secretion of testosterone [19]. On the other hand, low testosterone levels are believed to contribute to diabetes and insulin resistance, so these processes may form a vicious cycle [23].

It is intriguing that TT was not correlated with age or duration of diabetes, as this result is inconsistent with other research [11]. This may suggest a difference in late-onset hypogonadism. We also analyzed the relationship between TT and LH and FSH. We found that LH and FSH levels were decreased in the low testosterone groups, such that TT was positively correlated with LH and FSH. Compared with the high LH group, TT concentration decreased, and the incidence of hypogonadism was higher in the low LH group. Clinical studies have found that, in addition to low testosterone levels, 25% of males with type 2 diabetes also present with low levels of LH and FSH [24]. In addition, previous investigations have found that 33% of patients with hypogonadism also had LH and FSH levels that were significantly reduced [25]. Our results further confirm that diabetes patients present with hypogonadotropic hypogonadism.

In previous studies, however, the association between serum TT and gonadotropin was not clear in males with type 2 diabetes. It has been shown that using insulin to incubate hypothalamic neurons can promote the secretion of gonadotropin-releasing hormone (GnRH), but this effect is inhibited by insulin deficiency [26]. On the other hand, obesity can promote estrogen secretion and suppress hypothalamic GnRH production [27]. In addition, tumor necrosis factor-alpha, IL-1 beta, C-reactive protein, and other inflammatory factors can suppress the release of GnRH and LH in states of hyperglycemia [28, 29]. In sum, hyperglycemia or diabetes can influence the hypothalamus-pituitary-gonad axis to cause hypogonadism [25, 30].

Metabolic syndrome is the cooccurrence of clinical disorders including obesity, hypertension, hyperglycemia or diabetes, hypertriglyceridemia, low HDL-c, and other metabolic disorders. It is a major risk factor for type 2 diabetes and cardiovascular disease. Therefore, the relationship between metabolic syndrome and gonadal function merited further exploration [31, 32]. We found that as the number of risk factors increased, the serum TT level decreased gradually. This indicates that the more serious the metabolic disorder, the higher the risk of hypogonadism. In other words, there was a significant correlation between serum TT and metabolic syndrome. Although blood pressure, hypertriglyceridemia, and low HDL-c were not associated with hypogonadism individually, as components of metabolic syndrome, they may increase the risk of hypogonadism in patients with MS.

At present, diabetes with hypogonadism is commonly observed in the clinic, especially in male patients. The International Society for Andrology has recommended that patients do not need testosterone replacement therapy if TT levels are higher than 12 nmol/L. However, testosterone supplementation can benefit patients with TT levels less than 8 nmol/L [12]. Nonetheless, studies show that, despite the clinical benefits [33–35], testosterone replacement therapy still does not receive enough attention, resulting in many patients developing clinical symptoms to varying degrees. Thus, we need to assess gonadal function, confirm the diagnosis of hypogonadism as early as possible, and implement appropriate hormone replacement therapy to alleviate patients' clinical symptoms and improve the quality of life in those with type 2 diabetes.

There are some limitations to our study. First, this was a retrospective study; thus, we lacked a sexual dysfunction assessment scale, such as the International Index of Erectile Function or the Sexual Health Inventory for Men. Beyond low TT concentration, the symptoms of sexual dysfunction are an important aspect of male hypogonadism. Second, because free testosterone levels can reflect the extent of testosterone's biological activities, the free testosterone concentration should be used to assess hypogonadism, but the determination of free testosterone is difficult, so free testosterone is calculated frequently with formula in practical work. At last, we did not talk about the relationship between hypogonadism and income, education, smoking, and alcohol drinking.

## 5. Conclusion

In conclusion, in the present study, several risk factors of diabetes associated closely with hypogonadism. BMI, MS, HOMA-IR, and LH are independent risk factors for hypogonadism in male patients with type 2 diabetes.

## Figures and Tables

**Table 1:** Comparison of general data in the two groups.

	Low TT group	Normal TT group	*p*
*N*	75	138	
Age (years)	53.25 ± 11.21	50.26 ± 11.38	0.382
Duration (months)	49.32 ± 52.14	53.77 ± 45.24	0.576
BMI (kg/m^2^)	27.32 ± 4.53	23.31 ± 3.64	0.021
WC (cm)	92.22 ± 9.45	83.36 ± 11.51	0.048
SBP (mmHg)	152.32 ± 34.25	149.62 ± 36.83	0.072
DBP (mmHg)	95.23 ± 18.35	96.39 ± 16.52	0.343
Complications			
Diabetic nephropathy (%)	6 (8.0)	15 (10.8)	0.092
Diabetic retinopathy (%)	10 (14.3)	18 (13.0)	0.254
Diabetic neuropathy (%)	7 (9.3)	13 (9.4)	0.146
PAD (%)	12 (9.14)	17 (12.3)	0.096
CHD (%)	5 (6.7)	7 (5.1)	0.157
Cerebral infarction (%)	3 (4.0)	5 (3.6)	0.421
Comorbidities			
NAFLD (%)	35 (52.2)	14 (29.1)	0.025
Hypertension (%)	49 (65.3)	82 (59.4)	0.983
Hypertriglyceridemia (%)	36 (48.0)	55 (39.8)	0.062
Low HDL-c (%)	37 (49.3)	56 (40.5)	0.125
Hyperuricemia (%)	16 (21.3)	22 (15.9)	0.071
Overweight or obesity (%)	50 (66.7)	68 (49.3)	0.036

Data are presented as the mean ± SD or the number of subjects in each group with percentages given in parentheses. The percentages were calculated based on the number of patients with evaluable data rather than the total eligible population. BMI, body mass index; WC, waist circumference; SBP, systolic blood pressure; DBP, diastolic blood pressure; PAD, peripheral arterial disease; CHD, coronary heart disease; NAFLD, nonalcoholic fatty liver disease.

**Table 2:** Comparison of indicators of glucolipid and insulin in two groups.

	Low TT group	Normal TT group	*p*
*N*	75	138	
HbA1c (%)	9.16 ± 2.35	9.74 ± 2.54	0.187
FBG (mmol/L)	8.27 ± 234	8.46 ± 2.39	0.194
PBG (mmol/L)	15.15 ± 4.03	14.42 ± 4.13	0.153
TC (mmol/L)	4.57 ± 1.25	4.37 ± 1.03	0.091
TG (mmol/L)	2.38 ± 2.92	2.14 ± 1.76	0.077
LDL (mmol/L)	2.61 ± 0.62	2.52 ± 0.75	0.131
HDL (mmol/L)	1.06 ± 0.39	1.14 ± 0.35	0.096
FINS (*μ*IU/L)	9.98 ± 6.06	7.23 ± 7.32	0.048
PINS (*μ*IU/L)	48.43 ± 55.21	36.31 ± 46.35	0.051
HOMA-IR	3.72 ± 2.23	3.01 ± 2.08	0.035

Data are presented as the mean ± SD in each group. HbA1c, glycosylated hemoglobin; FBG, fasting glucose; PBG, 2 h postprandial blood glucose; TC, cholesterol; TG, triglyceride; LDL-c, low-density lipoprotein cholesterol; HDL-c, high-density lipoprotein cholesterol; FINS, fasting insulin; PINS, 2 h postprandial insulin; HOMA-IR, HOMA insulin resistance index.

**Table 3:** Comparison of sex hormone in two groups.

	Low TT group	Normal TT group	*p*
*N*	75	138	
TT (nmol/L)	9.25 ± 1.43	18.22 ± 5.30	0.000
SHBG (nmol/L)	22.37 ± 12.45	31.29 ± 23.36	0.012
P (nmol/L)	1.35 ± 0.58	1.21 ± 0.64	0.075
PRL (*μ*IU/L)	304.38 ± 97.35	296.37 ± 113.25	0.212
LH (mIU/L)	6.04 ± 3.52	8.56 ± 3.41	0.021
FSH (mIU/L)	6.86 ± 3.28	8.73 ± 5.36	0.047
E2 (pmol/L)	87.67 ± 53.39	93.26 ± 47.21	0.068
DHEA (*μ*mol/L)	6.17 ± 3.15	5.42 ± 2.81	0.083

Data are presented as the mean ± SD in each group. TT, total testosterone; SHBG, sex hormone binding globulin; P, progesterone; PRL, prolactin; LH, luteinizing hormone; FSH, follicle-stimulating hormone; E2, estradiol; DHEA, dehydroepiandrosterone.

**Table 4:** Logistic regression analysis for risk factor of hypogonadism.

	*β* value	SE	Wald	*p*	OR	95% CI
Age	0.124	0.013	5.211	0.063	1.109	0.201–1.015
Duration	0.103	0.056	6.35	0.271	1.058	0.546–1.021
BMI	0.112	0.032	6.011	0.016	1.315	1.034–1.216
WC	0.183	0.046	2.811	0.084	1.136	0.056–1.021
FBG	0.078	0.051	3.938	0.964	1.076	0.159–1.143
HbA1c	0.432	0.165	6.814	1.523	0.821	0.113–2.140
TG	0.118	0.105	3.103	0.219	1.013	0.104–1.239
HDL-c	0.204	0.076	7.043	0.051	1.079	0.126–1.335
FINS	0.318	0.270	6.375	0.089	1.028	0.096–1.062
HOMA-IR	0.123	0.046	5.745	0.036	1.246	1.045–1.238
LH	0.159	0.157	8.128	0.047	1.217	1.021–1.153
FSH	0.138	0.144	7.282	0.052	1.077	0.018–1.067
MS	0.043	0.021	5.128	0.039	1.258	1.012–1.129

Note: BMI, body mass index; WC, waist circumference; FBG, fasting glucose; HbA1c, glycosylated hemoglobin; TG, triglyceride; HDL-c, high-density lipoprotein cholesterol; FINS, fasting insulin; HOMA-IR, HOMA insulin resistance;  LH, luteinizing hormone; FSH, follicle-stimulating hormone; MS, metabolic syndrome.
